# Selected abstracts from the 9^th^ CDD MRC Conference: Genes vs Environment in Cancer

**DOI:** 10.1038/s41420-018-0129-3

**Published:** 2019-01-03

## Abstract

**Sponsorship:** Publication of this supplement was sponsored by the Medical Research Council Toxicology Unit (MRC-TU). All content was reviewed and approved by the Cell Death and Differentiation (CDD) Committee, which held full responsibility for the abstract selections.

## CDD 01 TRAP1 regulation of energy metabolism in cancer cells

### Danilo Swann Matassa^1^, Rosario Avolio^1^, Giuseppe Di Lorenzo^1^, Aleida Acampora^1^, Matteo Landriscina^2^, Franca Esposito^1^

#### ^*1*^*University of Naples Federico II, Via S. Pansini 5, 80131 Naples, Italy;*^*2*^*University of Foggia, Viale Pinto 1, 7100 Foggia, Italy*

Metabolic rewiring sustains all other cancer hallmarks, thus representing a key target for therapeutic interventions. In such a context, increasing roles are attributed to molecular chaperones, which are not just multifunctional proteins, but rather molecular hubs connecting different metabolic pathways. TRAP1 is a HSP90 molecular chaperones, involved in the regulation of energy metabolism in cancer cells. Unpublished data by our group suggest that TRAP1 regulation of cell metabolism is dependent on the translational control of mitochondrial proteins, including UQCRC2 subunit of respiratory complex III.

Interestingly, an unexpected inter- and intra-tumor metabolic heterogeneity strictly correlates to tumor outcome. Recent evidence suggests that TRAP1 is co-expressed with the majority of its client proteins in human colorectal carcinomas and hierarchical cluster analysis showed that the upregulation of TRAP1 and associated 6-protein signature identifies a cohort of metastatic colorectal carcinomas with a significantly shorter overall survival.

Conversely, TRAP1 is downregulated in specific tumors (i.e., ovarian, bladder and renal cancers) where its lower expression correlates with worst prognosis and chemoresistance. Presently, TRAP1 role in enhancing or suppressing oxidative phosphorylation and the effect of such regulation on tumor development and progression are controversial. To shed light on molecular mechanisms/pathways controlled by TRAP1 a whole genome gene expression analysis was performed in platinum sensitive ovarian cancer cells upon TRAP1 silencing. Preliminary analysis identified redox/metabolic detoxification and interferon signaling as two pathways modulated by TRAP1 levels. By overlapping differential gene expression data and coexpression analyses in ovarian cancer patients, we identified a short-list of 10 genes whose expression is significantly correlated to TRAP1. Those include genes involved in drug metabolism and cholesterol biosynthesis. Noteworthy, the latter has also been found aberrantly dysregulated in several platinum-resistant ovarian cancer cell lines in comparison to their matched sensitive counterparts. Altogether these studies candidate TRAP1 as promising drug target linking chemoresistance and metabolic addiction.

## CDD 02 The LATS2 tumor suppressor, a positive regulator of p53, inhibits hepatic cholesterol accumulation and prevents aggressive luminal B breast cancer

### Noa Furth^1^, Ron Rotkopf^1^, Irina Rivkin^1^, Anat Gershoni^1^, Scott L. Friedman^2^, Randy L. Johnson^3^, Moshe Oren^1^, Yael Aylon^1^

#### ^*1*^*The Weizmann Institute of Science; Rehovot, Israel;*^*2*^*Icahn School of Medicine at Mount Sinai; New York, NY, USA;*^*3*^*The University of Texas MD Anderson Cancer Center; Houston, TX, USA*

LATS2 is a core kinase of the Hippo tumor suppressor pathway. Previously, we demonstrated that LATS2 possesses functions that go beyond its canonical role in the Hippo pathway, some of which rely on its ability to bind and inhibit MDM2. In this way, LATS2 contributes to p53 activation in response to mitotic and oncogene-driven stress. Furthermore, a positive feedback loop between p53 and LATS2 is activated in response to such stresses. We have generated mice harboring a conditional allele of Lats2. When Lats2 is specifically knocked-out in the liver (Lats2delHep), mice develop spontaneous fatty liver disease due to their inability to inhibit SREBP. When challenged with excess dietary cholesterol, Lats2delHep mice manifest more severe liver damage than wild-type mice, in conjunction with attenuated p53 activation, suggesting that LATS2-p53 activation plays a long-term tissue-protective role in this setting. Similarly, mammary gland-specific deletion of Lats2 (Lats2delMam) in a mouse model for luminal B breast cancer augments tumor burden and aggressiveness. This is in line with analysis of human breast cancer mega-datasets, in which we observe that LATS2 (but not its paralog LATS1) mRNA is specifically downregulated in luminal B breast cancer, and this is positively correlated with elevated risk of relapse.

## CDD 03 Long noncoding RNA in Brain Cancer

### Barak Rotblat

#### *Ben-Gurion University of the Negev, Beer Sheva, Israel*

Genes whose products are long noncoding RNA are highly abundant in the human genome however, we know very little of the function or clinical relevance of the vast majority of these genes. We found that TP73-AS1, one such lncRNA, is clinically relevant in brain cancer and in glioblastoma (GBM) and medulloblastoma its high expression in tumors is correlated with poor patient outcome. Using gene silencing approach and tumor derived cell lines we found that TP73-AS1 promotes tumorigenicity in medulloblastoma and chemotherapy resistance in GBM. These findings bring forth TP73-AS1 as a promising prognostic and therapeutic target and the potential of lncRNA to play important roles brain cancer.

## CDD 04 MiR-205-5p promotes invasion and metastasis in breast cancer stem cells

### De Cola A^1^, Lamolinara A^1^, Lanuti P^1^, Rossi C^1^, Iezzi M^1^, Todaro M^2^_,_ De Laurenzi V^1^

#### ^*1*^*Center of Excellence on Aging and Translational Medicine (CeSi-Met), G. D’Annunzio University, Chieti-Pescara, Italy;*^*2*^*University of Palermo, DiBiMIS, Palermo, Italy*

Breast cancer stem cells, a subpopulation of tumor cells with stem-like properties, play a pivotal role in tumor growth and metastatic progression contributing to therapy resistance. Metastatic process is sustained by epithelial to mesenchymal transition (EMT) program by which epithelial cells convert into mesenchymal phenotype acquiring the ability to invade and disseminate from the primary tumor site to distant tissues. EMT is induced by several stimuli from the tumor microenvironment but also microRNAs can mediate it based on the activity on target genes. In this study we report that reduced level of miR-205-5p by Locked Nucleic Acids molecular approach (LNA), impairs metastatic potential and tumor progression of breast cancer stem cells, modulating the expression of EMT key transcription factors. Furthermore, miR-205 silencing attenuates breast cancer stem cells stemness phenotype, suggesting miR-205 as a novel target for breast cancer therapies.

## CDD 05 Birinapant augments the efficacy of Isolated limb perfusion in an animal model of extremity soft tissue sarcoma

### Kunzah Jamal^1^, Henry Smith^1^, Tencho Tenev^1^, Joan Kyula^1^, Victoria Roulstone^1^, Kevin Harrington^1^, Pascal Meier^1^

#### ^1^*Institute of Cancer Research, Mary-Jean Mitchell Green Building, Chester Beatty Laboratories, Fulham Road, London SW3 6JB, UK*

The addition of tumour necrosis factor (TNF) has significantly improved the efficacy of Isolated limb perfusion (ILP) for the treatment of extremity soft tissue sarcomas (ESTS). However, in most cases metastases occur limiting disease free survival. Birinapant is a bivalent SMAC mimetic (SM), which synergises with TNF and induces immunogenic cell death. Here we show that the addition of BIR to the established ILP regime significantly increases the survival of an orthotopic model of ESTS. The rat sarcoma cells, BN175, are moderately sensitive to the combination of TNF, melphalan and SM in vitro. Mechanistically, the death induced by TNF and SM is RIPK1 and capsase-8 dependent. FACS analysis of the SM treated tumours reveals increased FOXP3-expressing regulatory T (Treg) cells infiltration compared to the standard of care suggesting that the reoccurrence of disease could be due to an immune-suppressing environment. In fact, immune checkpoint blockage post SM treatment further increases survival compared to the standard of care. Furthermore, tumour cell lines derived from the SM/ILP treated cohort remain sensitive to SM *in vitro* suggesting that tumour re-occurrence is due to the initial treatment not killing off the tumour population. In accordance, treatment of BN175 cells with a novel TLR3 agonist riboxxol and SM potently kills BN175 *in vitro* suggesting that *in vivo*, riboxxol and SM could be applied systemically to treat recurring malignancies and prolong disease free survival.

Acknowledgment: KJ, HS contributed equally to this work.

## CDD 06 The lncRNA NORAD correlates with aggressive breast cancer subtypes and confers resistance to chemotherapy

### Beatriz Silva^1^, Catarina Alves-Vale^1^, Carla Pereira Gomes^1^, Sérgio Pires Marinho^1^, Bruno Bernardes de Jesus^1^

#### ^*1*^*Universidade de Lisboa, Av. Professor Egas Moniz, 1649-028 Lisboa, Portugal*

The recently discovered lncRNA NORAD is induced after DNA damage in a p53-dependent manner and plays a critical role in the maintenance of genomic stability, since interacts with Pumilio proteins, limiting the repression of their target mRNAs involved in mitosis, DNA repair and replication. Therefore, NORAD inactivation causes chromosomal instability, aneuploidy and tumorigenesis, suggesting that it functions as a tumor suppressive factor, as reported for liver cancer. However, studies performed in esophageal, breast, lung, pancreatic, bladder and colorectal cancers, reported that NORAD functions as an oncogenic factor. The contradictory results existent in the literature and poor characterization in breast cancer, motivated us to unravel the role of NORAD in this cancer type.

In this study, high NORAD expression is associated with aggressive breast cancer subtypes (MDA-MB-231, 436 and 468) (RT-qPCR) and poor relapse-free survival of patients (Kaplan-Meier Plotter). We selected the MDA-MB-231 cell line, and we observed that NORAD knockdown (LNA^TM^ GapmeRs and siRNAs) inhibits cell viability (alamarBlue^®^ reduction assay), migration (wound healing assay), arrests cells in S phase (flow cytometry), and sensitizes them to chemotherapy (doxorubicin) through apoptosis or mitotic catastrophe (alamarBlue^®^ reduction assay and flow cytometry). Relative to the mechanism of action, we observed that upon NORAD knockdown, the DNA damage induced by doxorubicin seems not to be signaled by γH2AX to the cell for the recruitment of DNA repair machinery (comet assay and western blot). The next step is to inactivate Pumilio proteins to see if γH2AX signaling is restored. In summary, NORAD functions as an oncogenic factor, confers resistance on cancer cells to chemotherapy and interferes with γH2AX signaling of the DNA damage, in breast cancer.

## CDD 07 Human endogenous retroviruses are responsive to microenvironmental changes and are associated to phenotype switching in different types of tumor cells

### Matteucci C^1^, Balestrieri E^1^, Giovinazzo A^1^, Gambacurta A^1^, Argaw-Denboba A^1, 2^, Cipriani C^1^, Petrone V^1^, Sinibaldi-Vallebona P^1, 3^

#### ^*1*^*University of Rome “Tor Vergata”, Italy;*^*2*^*European Molecular Biology Laboratory (EMBL), Adriano Buzzati-Traverso Campus, Monterotondo, Rome, Italy;*^*3*^*Institute of Translational Pharmacology, National Research Council, Rome, Italy*

Human endogenous retroviruses (HERVs) are remnants of ancestral exogenous retroviruses infections and comprise about the 8% of human genome. HERVs are recognized to play a role in the carcinogenesis process, as they have been associated with cancer cells aggressiveness and a worsening prognosis in some cancer patients. Our research group is dedicated in studying the involvement of HERVs in complex diseases as genomic elements able to respond to external stimuli and therefore relevant in the crosstalk between cell and environment. In this scenario, we have already demonstrated that HERV-K activation under microenvironmental stress conditions is strictly required in human melanoma cells to acquire an aggressive phenotype and to expand a subpopulation of cancer cells with stemness features. Our hypothesis is that HERVs activation may represent a mechanism common to different types of tumour, needed to respond to stress due to changes in the tumor microenvironment and to support tumor aggressiveness. In this view, we analysed the expression of selected HERV families in different type of tumour cells, such as melanoma, hepatocellular carcinoma, breast and colon cancer, subjected to microenvironmental modifications, and we evaluated the correlation with the acquisition of stemness and aggressiveness features. In response to environmental modifications by exposure to different culture conditions, we demonstrated that the induction of HERV-K and HERV-H expression is associated with modifications in cellular morphology, generation of sphere-like non-adherent cell aggregates and an increased expression of the stem cell marker CD133. Our findings highlight HERVs as genomic elements particularly responsive to tumor microenviromental changes and to aggressiveness features of cancer cells, suggesting a role as potential targets for therapy.

## CDD 08 Chronic exposure of Bisphenol A induces epithelial to mesenchymal transition in LNCaP cells via AR/PGC-1ɑ/PPAR-γ signalling

### Vipendra Kumar Singh^1, 2^, Mohammad Imran Ansari^1, 2^, Yogeshwer Shukla^1, 2^, Pradeep Kumar Sharma^1, 2^

#### ^*1*^*Food Toxicology Division, Food Drug and Chemical Toxicology Group, CSIR-Indian Institute of Toxicology Research 31, Vishvigyan Bhawan Mahatma Gandhi Marg, Lucknow-226001, India;*^*2*^*Academy of Scientific and Innovative Research (AcSIR), CSIR-Indian Institute of Toxicology Research Campus, Lucknow 226001, Uttar Pradesh, India*

Bisphenol A is a potential endocrine disruptor and is known for inducing transformation in breast cells via estrogenic effects. However, its androgenic potential and androgen dependent transformations are poorly known. In the present study, we show that the chronic exposure of environmentally relevant dose of Bisphenol A (50 nM per day for 150 days) enhances the expression of androgen receptor (AR) in LNCaP cells. Chronic exposure of BPA significantly enhanced proliferation, colony formation ability of LNCaP cells. BPA also induced epithelial to mesenchymal transition (EMTs) in LNCaP cells as demonstrated by enhanced expression of genes and proteins of various EMT and metastatic markers. Moreover, it induces genomic instability via enhanced micronuclei formation (MN) and DNA double-stranded breaks (DSBs). Also, the chronic exposure of BPA augmented mitochondrial biogenesis, ATP synthesis and fatty acid synthesis in LNCaP cells. Most interestingly, BPA up regulates the expression of PPAR-γ and transcriptional co-activator of PPAR-γ (PGC-1ɑ) in LNCaP cells in a time-dependent manner. Our observations demonstrate the androgenic potential of BPA and its transforming ability via AR/PGC-1ɑ/PPAR-γ signalling that regulates epithelial to mesenchymal transition to induce metastasis in LNCaP cells.

## CDD 09Patulin exposure induces TGF-β mediated Epithelial to Mesenchymal Transition in renal epithelial cells through up-regulation of slug/ snail genes

### Saurabh Pal^1, 2^, Neha Singh^1, 2^, Megha Bansal^1, 2^, Kausar Mahmood Ansari^1^

#### ^*1*^*CSIR- Indian Institute of Toxicology Research 31, Vishvigyan Bhawan, Mahatma Gandhi Marg, Lucknow-226001, India;*^*2*^*Academy of Scientific and Innovative Research (AcSIR), CSIR-Indian Institute of Toxicology Research Campus, Lucknow 226001, Uttar Pradesh, India*

Patulin (PAT) is a secondary metabolite of fungal origin produced by certain species of *Aspergillus*, *Byssochlamys*, and *Penicillium* that is commonly found in rotten fruits and vegetables, principally in apples and apple-based products. The toxic effect of PAT is well known and its role in tumor initiation has been suspected for a long time. The present study elucidates a novel mechanism through which the environmentally relevant low dose exposure of PAT regulates the epithelial to mesenchymal transition (EMT) in normal renal epithelial cells by augmenting the levels of slug and snail proteins, mediated by transforming growth factor-β (TGF-β) through the canonical Smad-dependent signaling pathway. Additionally, the paracrine effects of TGF-β were reported to induce EMT via induction of c- jun/AP-1 transcription factors in bystander cells. Therefore, PAT was reported to induce EMT phenotype through TGF-β/Smad/Snail signaling via c-Jun/AP-1 activation. Various mesenchymal, as well as cellular proliferation markers expression, were checked by using RT-PCR, western blot and chromatin immunoprecipitation (ChIP) assay to assess the protein-protein and protein DNA interaction. Our study demonstrated that the low dose chronic exposure of PAT (100 nM) regulates epithelial to mesenchymal transition in normal kidney cells. This study provides insights into the molecular mechanism of EMT induction in PAT exposed normal cells, which may guide future studies to the target upon its therapeutic intervention. Moreover, this study also provides considerable evidence that environmental exposure of PAT may be hazardous for human health.

## CDD 10 Meiotic/Mitotic microtubule bundler DLGAP5, docetaxel and the androgen-regulated cell cycle: a three-way synergy

### Kay Hewit^1^, Emma Sandilands^1^, Daniel James^2^, Hing Leung^1, 2^, David Bryant^1, 2^, Emma Shanks^2^, Elke Markert^1^

#### ^*1*^*University of Glasgow, Glasgow, UK; CRUK Beatson Institute, Glasgow, UK*

DLGAP5, or HURP, is expressed during mitosis on the kinetochores and the chromatin side of the mitotic spindles. It is essential during meiosis, where it bundles microtubule and controls the number of microtubule organizing centres; loss of the gene at this stage leads to infertility. Its knockdown in human cell lines however does not seem to cause dramatic effects on viability, as other factors organize microtubule sufficiently during mitosis. Here we show that in prostate cancer cells, knockdown of DLGAP5 sensitizes cells significantly to treatment by the microtubule-targeting agent docetaxel, however, surprisingly, in an androgen-dependent manner. Specifically, androgen-dependent LNCaP cells showed a significant synergy between the knockdown of DLGAP5 and docetaxel, whereas androgen-independent DU145, PC3 and androgen-desensitized LNCaP-AI cells did not. Androgen receptor inhibition in LNCaP cells attenuated the synergy effect. The data suggest that the androgen-regulated cell cycle has specific vulnerabilities under docetaxel that might have therapeutic implications, particularly in the light of earlier applications of chemotherapy in prostate cancer.

